# Derivation and validation of a simple, accurate and robust prediction rule for risk of mortality in patients with *Clostridium difficile* infection

**DOI:** 10.1186/1471-2334-13-316

**Published:** 2013-07-12

**Authors:** Emma Butt, Jane AH Foster, Edward Keedwell, Julia EA Bell, Richard W Titball, Aneel Bhangu, Stephen L Michell, Ray Sheridan

**Affiliations:** 1 Biosciences, College of Life and Environmental Sciences, Geoffrey Pope Building, University of Exeter, Stocker Road, Exeter EX4 4QD, UK; 2 Diabetes and Vascular Medicine Research, Institute of Biomedical and Clinical Science, Peninsula Medical School, Barrack Road, Exeter EX2 5AX, UK; 3 College of Engineering, Mathematics and Physical Sciences, Harrison Building, University of Exeter, North Park Road, Exeter EX4 4QF, UK; 4 Geriatrics and General (Internal) Medicine, South West Peninsula Geriatrics Training Programme, William Wright House, Royal Devon and Exeter NHS Foundation Trust, RD&E Hospital, Barrack Road, Exeter, Devon EX2 5DW, UK; 5 General Surgery Registrar Rotation, West Midlands Deanery, Edgbaston, Birmingham B16 9RG, UK; 6 William Wright House, Royal Devon and Exeter NHS Foundation Trust, RD&E Hospital, Barrack Road, Exeter, Devon EX2 5DW, UK

**Keywords:** All-cause mortality, *Clostridium difficile* related mortality, Non-*Clostridium difficile* related mortality, Clinical prediction rule

## Abstract

**Background:**

*Clostridium difficile* infection poses a significant healthcare burden. However, the derivation of a simple, evidence based prediction rule to assist patient management has not yet been described.

This study aimed to identify such a prediction rule to stratify hospital inpatients according to risk of all-cause mortality, at initial diagnosis of infection.

**Method:**

Univariate, multivariate and decision tree procedures were used to deduce a prediction rule from over 186 variables; retrospectively collated from clinical data for 213 patients. The resulting prediction rule was validated on independent data from a cohort of 158 patients described by Bhangu *et al*. (Colorectal Disease, 12(3):241-246, 2010).

**Results:**

Serum albumin levels (g/L) (P = 0.001), respiratory rate (resps /min) (P = 0.002), C-reactive protein (mg/L) (P = 0.034) and white cell count (mcL) (P = 0.049) were predictors of all-cause mortality. Threshold levels of serum albumin ≤ 24.5 g/L, C- reactive protein >228 mg/L, respiratory rate >17 resps/min and white cell count >12 × 10^3^ mcL were associated with an increased risk of all-cause mortality. A simple four variable prediction rule was devised based on these threshold levels and when tested on the initial data, yield an area under the curve score of 0.754 (P < 0.001) using receiver operating characteristics. The prediction rule was then evaluated using independent data, and yield an area under the curve score of 0.653 (P = 0.001).

**Conclusions:**

Four easily measurable clinical variables can be used to assess the risk of mortality of patients with *Clostridium difficile* infection and remains robust with respect to independent data.

## Background

*Clostridium difficile* (*C. difficile*) is an anaerobic, spore forming, rod shaped bacterium, and is a prevalent Healthcare Care Associated Infection (HCAI). Furthermore, antibiotic resistant strains of *C. difficile* are a growing problem in the healthcare system [1]. Recent *C. difficile* epidemics have been caused by hypervirulent strains which became resistant to the fluoroquinolones soon after their introduction into the healthcare setting [2]. Current UK approved therapy for *C. difficile* infection is oral vancomycin, oral or intravenous metronidazole, or fidaxomicin among other treatment regimens [3]. According to the National Office of Statistics, *C. difficile* related deaths accounted for 1.1% of all deaths in England and Wales between 2006 and 2010 with patients over 65 years having a particularly high incidence of mortality [4]. Further difficulties are faced when trying to predict recurrence, which occurs in approximately 20% of patients following withdrawal of antibiotics and this makes subsequent therapeutic choice more complex [5]. As well as identifying significant risk factors for development of *C. difficile* infection (CDI) it is also of clinical importance to produce a valid system to describe severity and predict morbidity and mortality in order to provide effective treatment regimes.

### Clinical prediction rules

There have been few studies which have generated simple prediction rules for risk of mortality for patients with HCAIs, and more specifically CDI. As discussed in a recent BMJ article by Adams *et al*. (2012) [6] clinical prediction rules that are simple to use in the busy hospital setting and that add prognostic value to clinical evaluation should be considered in more areas, as is highlighted by the successful implementation of scoring systems such as CURB-65 in patients with community acquired pneumonia [7]. The implementation of a disease specific score such as the CURB-65 clinical prediction rule has helped predict mortality risk and guide treatment options in patients with community acquired pneumonia. The merits of CURB-65 clinical prediction rule are that it relies upon only a few variables, and is now a clinically wide-spread tool easily used non-specialists [7]. Thus, the aim of this study was to develop a similar simple clinical prediction rule that would help in a similar manner in patients with CDI, and could easily be applied by non-specialist or a junior doctor pending review by a more experienced clinician. The identification of those patients with CDI who are more at risk from mortality could facilitate more targeted and intensive treatment regimens [8] and facilitate bedside clinical decision making [9].

In 2012 a systematic review of prediction rules for outcomes in CDI patients found only a small number of studies (N = 13) which had derived rules for outcomes such as severity of disease (N = 5), mortality, (N = 5), recurrence, (N = 2) and response to therapy (N = 1) of which, only two rules had been tested on a validation cohort [10]. A recent publication by Bhangu *et al*. (2010) [11] evaluated the use of significant clinical variables identified by univariate and multivariate tests, for the development of a scoring system to identify patients with CDI who may be more at risk from death during the course of infection. This study identified six clinical variables and associated the following threshold values in a cohort of 158 patients; age ≥ 80, clinical disease severity, white cell count ≥ 20 × 10^3^ mcL or C-Reactive protein ≥ 150 mg/L, urea ≥ 15 mmol/L and serum albumin ≤ 20 g/L which could be used in combination to score the relative risk of death in patients with CDI. By assigning one point to each variable and using a score from 0–5 (evaluated within the first 72 hrs of a toxin positive stool sample) the risk of death increased as the score increased. However, some of the variables used in the scoring system are themselves derived from other observations, making the system more complex and more heavily reliant on clinician interpretation. Many of the other rules [12-14] mentioned in the meta analysis [10] may also be underutilised in clinical practice due to being over complicated with too many variables. Simple, accurate prediction rules, relying on few variables, for identifying the risk of mortality in patients with CDI; that can be applied to a case within a clinical setting and be used effectively to monitor patients’ treatment regimes, are of great interest to healthcare system. Yet, as described above there is little published literature regarding the derivation and validation of clinical prediction rules for CDI patients, further emphasising the necessity of being able to readily identify patients who are more at risk from death, in a simple, clear and accurate manner.

### Study objective

This retrospective cohort study using an independent validation cohort has been designed to obtain a simple prediction rule to stratify hospital inpatients at initial diagnosis of CDI according to risk of mortality, using univariate, multivariate and decision tree procedures.

## Method

Royal Devon and Exeter Hospital (RD&E) NHS Trust is a 49 ward, 797 inpatient-bed hospital. The mean CDI rate was 6.55 hospital apportioned cases per 10,000 bed days from the period March 2007- April 2009 as reported by the Health Protection Agency. The national average across England for the same period was 7.4 hospital apportioned cases per 10,000 bed days. The range of incidences of trust apportioned cases of CDI per 10000 bed days derived from 165 hospitals in England was 0.1-14.6 during the same period.

Inclusion criteria for this study were symptomatic patients with a *C. difficile* toxin A/B positive stool sample using the TechLab® C.diff Quik Chek Complete™ Enzyme linked Immuno-assay (Alere Ltd, UK), who were transferred to the specialised *C. difficile* cohort ward between 2007 and 2009. This cohort ward is a 19 bed ward with 7 double doored, single occupancy side rooms and cohorted bays with closed doors. Patients were managed by a multi-disciplinary specialist team including a physician, microbiologist, antibiotic pharmacist, infection control nurse and physiotherapists. Having identified the study population, the clinical notes were reviewed for the admission period during which the positive toxin result was confirmed. Information parameters collected and used in analysis included: biographical details, concurrent medication (chemotherapy agents, gastro-suppressants and steroids) co-morbidities, pre-admission place of residence, pre-admission circumstance, antibiotic administration in the previous two months, faecal calprotectin results, CDI treatment regimes, routine blood tests and observations at time of diagnosis. Clinical blood measurements and routine observations i.e. blood pressure, respiratory rate etc. were those first taken within 48 hours of a *C. difficile* toxin A/B positive test. The definition of all-cause mortality in this study is; patients who had died from CDI and non-CDI related causes as an inpatient, or had been discharged and have then died ≤ 30 days. All patients were followed for a minimum of 30 days post discharge and the total number of patients in this study was 213. The total number of cases for this study was 245, which included 29/213 patients with multiple *C. difficile* infections (CDIs). This project did not require ethics approval and it was clarified during scientific review that this is a retrospective study and data would be anonymised and collected as part of routine care.

### Statistical analysis

While the outcome of interest was all-cause mortality, statistical analysis was also used to deduce if there were means and medians of variables that were more significantly associated with either CDI or non-CDI related mortality versus those who survived (comparator group). Univariate and multivariate discovery of variables significantly associated with CDI and non-CDI related mortality was conducted on one set of patient (N = 213) data. For mortality this included one set of data from those with recurrent CDI, based on the last case entry, to account for those which may have had a worse outcome on subsequent infections. The data was anonymised and input into PASW Statistics 18 (SPSS Inc, IBM, USA). Nominal variables were converted into binary format unless dates were used as an input value.

All variables (Additional file 1) were then tested for normality for the outcome measure all-cause mortality using the Shapiro-Wilk test, where a P > 0.05 value was associated with normally distributed data.

All the demographic and baseline clinical characteristic data outlined above and in the supplementary data (Additional file 1), was used in univariate and multivariate analysis to identify variables significantly associated with mortality. All variables were tested for significance in relation to the outcome measures by independent median tests, One-Way ANOVA and Chi-squared (χ^2^) tests. Variables which remained statistically significant after Bonferroni correction (where P = 0.05/N; N = number of variables being tested on a test by test basis) were used in the regression model.

Variables independently associated with CDI related mortality and non-CDI related mortality versus the comparator group, were subject to multinomial logistic analysis on a patient basis. All significant variables were entered using a block entry method. Any missing data was excluded on a test by test basis and some data was not included in the analysis, to leave a portion of the data as ‘unseen’ to avoid overfitting in the classification and regression tree (CRT) analysis. These algorithms, whilst powerful, can overfit their training data and develop models that are overspecialised to one dataset. The use of a separate testing set to reduce this effect is standard practice in classification research.

### Model derivation

For the purpose of prediction rule derivation in this study, the mortality outcome was grouped into two categories (died and survived). The prediction rule was derived by inputting all 245 cases of data (patients with primary only and recurrent CDI) into the analysis, which was then split into training and test data (50:50) in order that a sufficient amount of data was captured in each portion. A decision tree classification model was used to predict values of a dependent (target) variable based on values of independent (predictor) variables (SPSS Inc). All results for derivation of threshold values are reported on test data. (Model criteria can be found in the Additional file 1).

Variables and their respective threshold values which were found during multivariate analysis and test data of classification analysis were assigned a score to create a prediction rule which was then applied to the derivation and validation cohort for all applicable cases.

### Model validation

Independent data from 158 patients with CDI obtained from a UK teaching hospital, as described by the Bhangu *et al*. (2010) study [11] was used to validate the prediction rule generated in the derivation cohort. The prediction rule, excluding the variable respiratory rate (due to lack of data for this measure) was evaluated in each cohort by receiver operating characteristic (ROC) curves.

## Results

### Derivation cohort demographics

54% (115) of the population were female. 71% (174) of cases were admitted for acute medical care, 10% (25) of cases were admitted due to community acquired CDI and the remaining cases were admitted for; elective surgery (7), emergency surgery (15), trauma (12), planned procedures (1) or renal care (11)*.* 78% (191) cases were originally admitted from the home environment, 13.5% (33) of cases were originally admitted from another hospital and 8.6% (21) of cases were originally admitted from long term care facilities. All but three cases (who were later transferred to the intensive care ward) were admitted and treated in the *Clostridium difficile* cohort ward.

### Mortality

For this study cohort, 76% (162) of patients survived to discharge with the remaining 24% (51) either surviving to discharge then dying within 30 days (40), or dying within 30 days of admission (11) (all-cause mortality). The overall mortality rate for this cohort was 24% and CDI related mortality was 12.6%. Of the 51 patients who died at any stage after admission 53% (27) of these death certificates had *C. difficile* in part one or two according to the department of health guidelines [15] (CDI related mortality). Of these 27 deaths, 9 (33%) were due to CDI (listed in part one of the death certificate, according to the department of health report [15]) and 18 (66%) were not directly due to CDI but it was mentioned on the death certificate (listed in part two of the death certificate, according to the department of health report [15]). The remaining 24 deaths were not attributed in any way to CDI (non-CDI related mortality).

### Severity of infection

Severity of infection was noted for all cases (using local Department of Health guidelines [15]. There were 31.4% (77) cases of mild infection, 13.9% (34) cases of moderate infection, 47.3% (116) cases of severe infection and 7.3% (18) cases of life threatening infection. 29 patients had >1 CDI which gives a 14% recurrence rate. Further patient demographics can be seen in Additional file 1.

### Prediction rule derivation

#### Univariate and multivariate analysis

Results of Bonferroni post hoc analysis after One-Way ANOVA analysis revealed that there were significant differences in serum albumin means (see Additional file 1, for mean and median level pair wise comparisons) between the survival group and the groups; CDI related mortality (P = < 0.001) and non-CDI related mortality (P = 0.011), whilst Independent Samples K-median tests determined that the difference in median respiratory rate across the groups was significantly different (χ^2^(*df =* 2) =11.8; P = 0.003) from the grand median (16 resps/min). The difference in median C-Reactive protein (CRP) levels across the groups was determined to be significantly different (χ^2^(*df =* 2) =14.2; P = 0.001) from the grand median (89.50 mg/L), and the difference in median white cell counts (WCC) across the groups was determined to be significantly different (χ^2^(*df =* 2) =11.4; P = 0.003) from the grand median (12 × 10^3^ mcL).

Variables independently associated with the outcome all-cause mortality (survival group [N = 124], versus the group; CDI related mortality [N = 19], and non-CDI related mortality [N = 18]) were subject to multinomial logistic analysis on a patient basis. All significant variables were entered using a block entry method. Results revealed that the variables serum albumin level (P = 0.001), respiratory rate (P = 0.002), CRP (P = 0.034) and WCC (P = 0.049) significantly contributed to the model. Further breakdown of the model (Additional file 1) revealed that the variables respiratory rate and serum albumin were significant predictors for both non-CDI related mortality (P = 0.007; OR 1.186 and P = 0.004; OR 0.844) and CDI related mortality (P = 0.003; OR 1.222 and P = 0.001; OR 0.801), whilst CRP (P = 0.020; OR 1.009) and WCC (P = 0.025; OR 1.046) remained specifically statistically significant for the group CDI related mortality. The model showed an overall classification of 80.7%.

### Decision tree classification results

Due to the small number of patients in both the CDI-related mortality group and the non-CDI related mortality groups, for the purpose of decision tree classification analysis, these groups were merged into the single category outcome; *died*. All the variables showing significant association to both CDI-related and non-CDI related mortality were entered in a decision tree model to look for the measurements at which the tree partitioned the data into the outcome groups *died* and *survived*. The model used all four variables to classify test data (N = 120) and the overall accuracy of the model at classifying the data was 75%. The model accurately predicted 47% of cases into the outcome; *died*, and 80% of the survival outcome cases correctly using these four variables (Additional file 1). Inaccuracy in the test data may be due to the fact that there were only a small percentage of cases for the outcome, *died*.

Decision tree rules (not shown here) revealed that low serum albumin (≤ 24.5 g/L) and/or high CRP levels (> 228 mg/L) were both indicated in the increased probability of death as an outcome, however, in patients with serum albumin levels > 24.5 g/L and CRP levels > 228 mg/L, increased WCC (>12 × 10^3^ mcL) and increased respiratory rate (>17 resps/min) were all indicated in the increased probability of death as an outcome.

### Prediction rule

Based on the threshold levels indicated by the classification model, a prediction rule was then developed (Table 1) which assigned 1 point for a serum albumin level ≤ 24.5 g/L, 1 point for a CRP level > 228 mg/L and 1 point for a combination of WCC >12 × 10^3^ mcL and respiratory rate > 17 resps/min as this proved more discriminatory than either value alone. The summation of any combination of these variables as seen in Table 1, resulted in a score from 0–3 which could classify the cases into the group *survived* or *died*, in the all-cause mortality outcome. This prediction rule was applied to all the cases and cross tabulated with the all-cause mortality outcome. The results can be seen in Table 2 and Table 3 and showed that an increasing score (0, 1, 2, 3) increases the risk of mortality in patients with CDI.

**Table 1:** Summary of the prediction rule

**Variables**	
•	1 point for a Serum Albumin level ≤ 24.5 (g/L)
•	1 point for a CRP level > 228 (mg/L)
•	1 point for a combination of WCC > 12 (mcL) and respiratory rate > 17 (resps/min)

**Table 2:** Cross tabulation of score for risk of mortality against the actual mortality outcome

		**Score for mortality**				**Total**
		**0**	**1**	**2**	**3**	
**Died**	**Count**	16	21	10	3	50
	**(%) within Score for Mortality**	9.5	36.8	66.7	100.0	20.5
**Survived**	**Count**	153	36	5	0	194
	**(%) within Score for Mortality**	90.5	63.2	33.3	.0	79.5
**Total**	**Count**	169	57	15	3	244*
	**(%) within Score for Mortality**	100.0	100.0	100.0	100.0	100.0

*One missing case due to lack of data for all variables.

**Table 3:** Summary of mortality risk with increasing prediction rule score in the derivation cohort

**Score**	**Mortality risk (%)**	**Count (number of cases)**
**0**	9.5	16/169
**1**	36.8	21/57
**2**	66.7	10/15
**3**	100	3/3

As can be seen from Table 3, there is a linear increase in mortality risk in relation to the score progression from 0 to 3 points. A key point of the system is that the mortality risk for 0-scored patients is approximately half of the mean risk in the cohort as a whole.

### Prediction rule validation

The prediction rule shown in Table 1 was validated on independent data from 158 patients with CDI obtained from a UK teaching hospital as described by Bhangu *et al*. (2010) [11]. Respiratory rate data was not available for this cohort and so the prediction rule as derived in this study was run again omitting respiratory rate as a predictor, thus 1 full point was allocated to WCC >12 × 10^3^ mcL. The simplified prediction rule was tested on both data sets, and compared to the rule derived in the Bhangu *et al*. [11] study, and results can be seen in Table 4.

**Table 4:** **Prediction rule scoring system (excluding respiratory rate) tested on derivation cohort and validation cohort vs. the prediction rule derived by Bhangu *et al. ***[11]

	**Prediction rule risk score on derivation cohort (N = 244)**		**Prediction rule risk score on validation cohort (N = 154)**		**Bhangu *et al. ***[11]**prediction rule (N = 151)**		
**Score**	**Mortality risk**	**Count (number of cases)**	**Mortality risk**	**Count (number of cases)**	**Score**	**Count (number of cases)**	**Mortality risk**
**0**	10.4%	13/125	20.9%	9/43	**0-1**	19/86	22% (Low)
**1**	23.3%	20/86	37.1%	23/62	**2-3**	31/56	55% (medium)
**2**	42.9%	12/28	54.3%	25/46	**4-5**	8/9	89% (high)
**3**	100%	5/5	66.7%	2/3			

**Figure 1:**
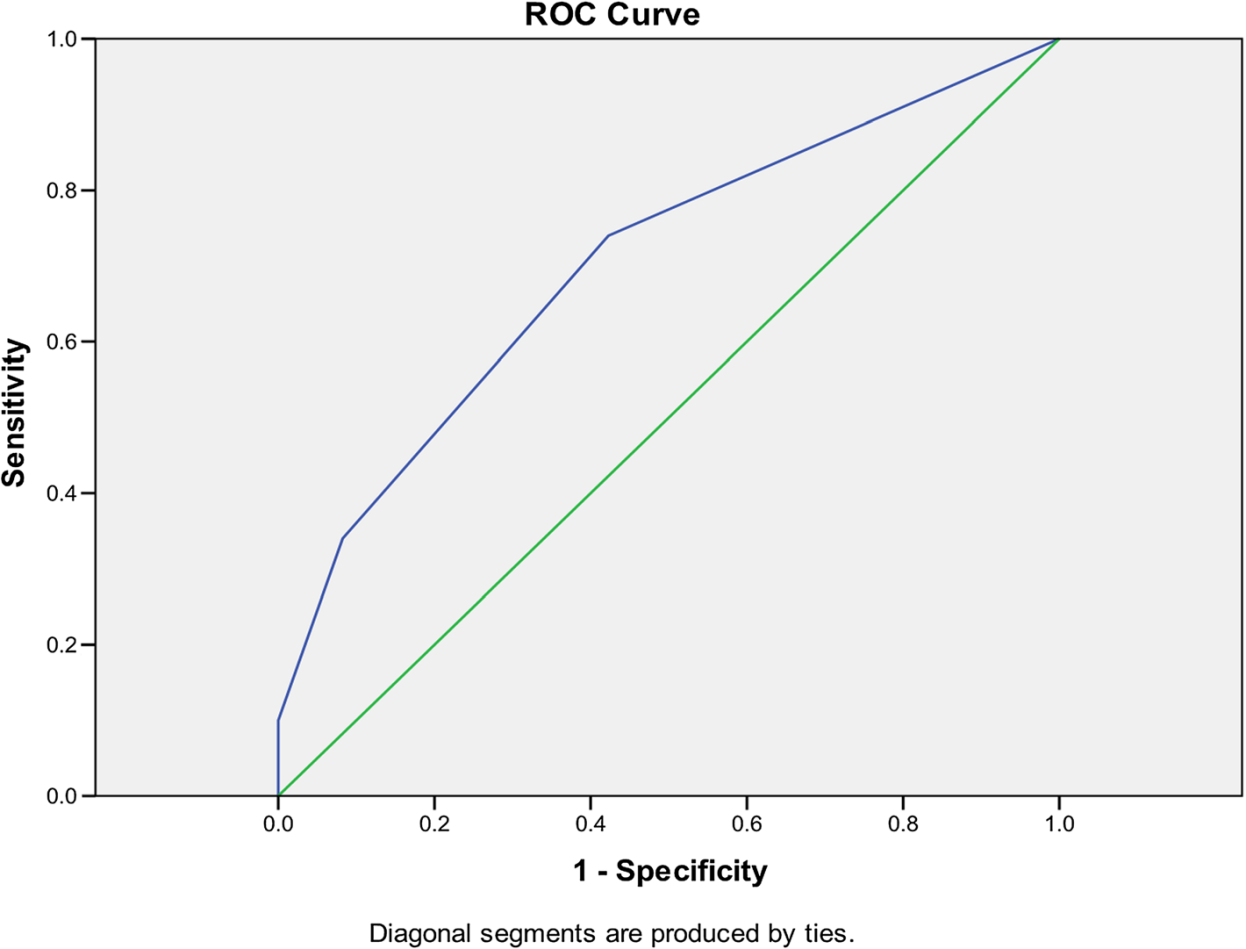
ROC curve for prediction rule (excluding respiratory rate) in the derivation cohort (AUC = 0.704; P < 0.001; 496 95% CI: 0.619-0.790).

**Figure 2:**
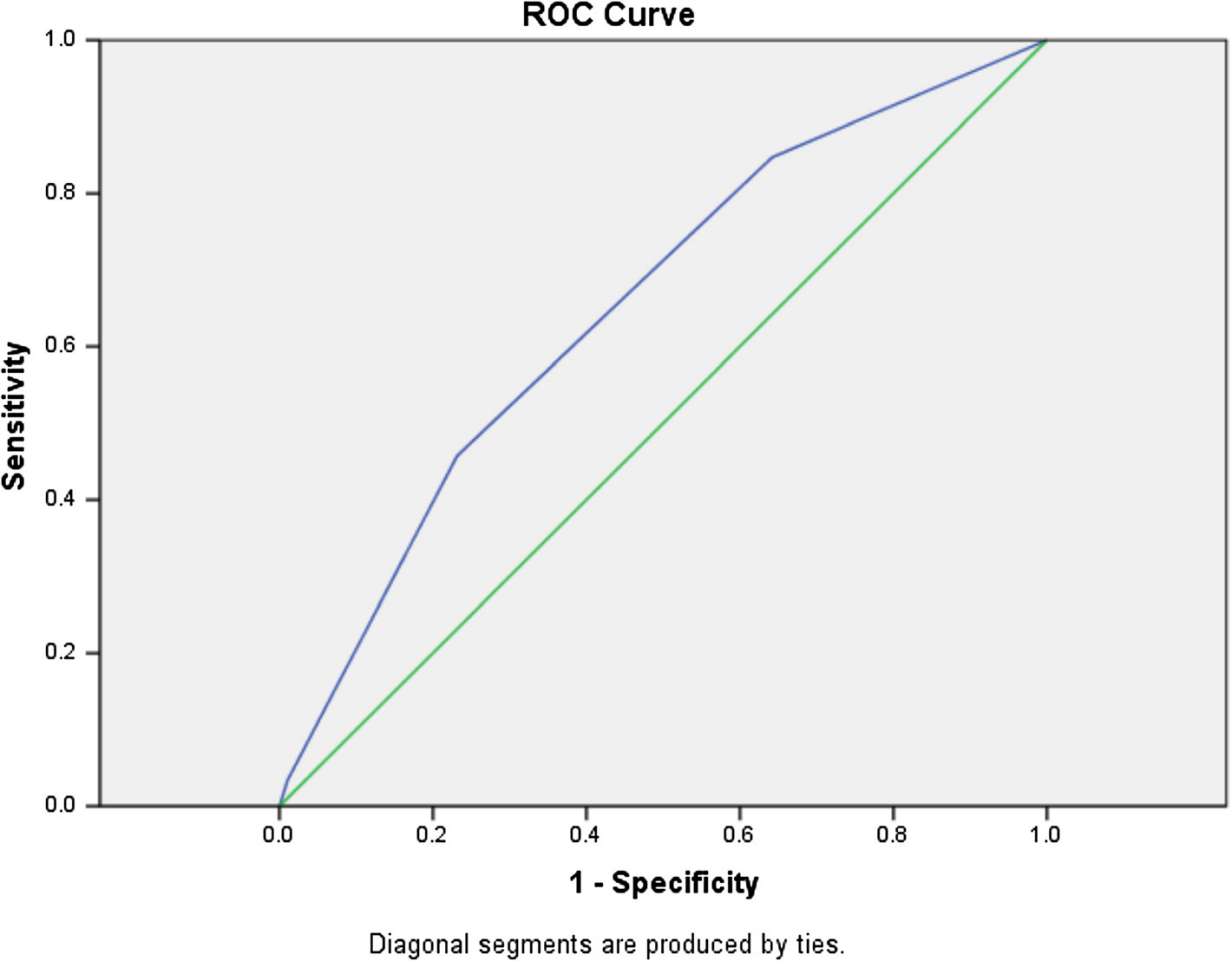
ROC curve for prediction rule (excluding respiratory rate) in the validation cohort (AUC = 0.653; P = 0.001; 95%; 502 CI: 0.565-0.741).

ROC curves were used to evaluate the original prediction rule and the prediction rule (excluding respiratory rate) on both the derivation and validation cohort. The original prediction rule yield an area under the curve (AUC) in the derivation cohort of 0.754 (P < 0.001; 95% CI: 0.670-0.837, data not shown) and the simplified prediction rule (excluding respiratory rate) tested in the derivation cohort (Figure 1) resulted in an AUC of 0.704 (P < 0.001; 95% CI: 0.619-0.790). The AUC of the simplified prediction rule was reduced by a further 5% to 0.653 (P = 0.001 95% CI: 0.565-0.741), when tested in the validation cohort (Figure 2) but remained statistically significant thus demonstrating the robustness of the prediction rule to new data.

## Discussion

### The prediction rule

This large retrospective cohort study has identified four easily measurable clinical variables: serum albumin level ≤ 24.5 g/L, CRP level >228 mg/L and a combination of WCC >12 × 10^3^ mcL and respiratory rate >17 resps/min (as this proved more discriminatory than either value alone) that if measured within or around 48 hours of CDI diagnosis, are capable of predicting the risk of mortality in patients with CDI. This prediction rule has been validated through an internal split sample procedure and on an independent cohort, and the variables are robust with respect to clinical threshold levels identified in other studies. These four variables can accurately assess the risk of mortality in patients with CDI and are not themselves defined by other parameters.

This simple prediction rule is more likely to be of practical use in the clinical setting than previously developed more complicated prediction rules, which have yet to become part of clinical practice. For example, the study conducted by Bhangu *et al.* (2010) [11] relied on six variables which could readily be measured, but one variable; severity of disease, is further defined by three more variables including sepsis, peritonitis and ≥ 10 episodes of diarrhoea in 24 hrs. The diagnosis of sepsis is further defined by the presence of diarrhoea with at least two other parameters that could include tachycardia (≥ 90 bpm), pyrexia (temperature ≥ 38°C), tachypnea (≥ 20 breaths per minute) or new onset hypotension. The addition of all these clinical parameters requires a more complicated and prolonged analysis than can be undertaken in the time constraints of a busy ward round.

The method used in this study allowed a classifier model to use a portion of data in training to generate a rule, which was then validated on a test data set, providing an unbiased model with greater accuracy. This study is unique in that it has used a decision tree model to evaluate threshold values for significant variables identified in multinomial logistic regression. A recent publication by Adams and Leveson (2012) [6] states that rules generated in this manner are generally easily understood and translatable into everyday clinical practices, but could lose accuracy if too little information is used to generate the rule. However, we do not feel this was the case during this study due to the comprehensive set of variables which were analysed.

### Interpretation

The four variables; serum albumin (g/L) (P = 0.001), CRP (mg/L) (P = 0.020), WCC ( × 10^3^ mcL) (P = 0.025) and respiratory rate (resps/min), (P = 0.003) were identified by univariate and multivariate analysis as being significant predictors of all-cause mortality in patients with CDI (Additional file 1). These are all clinical measurements that could be readily obtained at the time of CDI diagnosis and are likely to be taken routinely from hospitalised patients with symptoms suggestive of CDI, which makes this prediction rule very accessible in a clinical setting. A meta-analysis by Bloomfield *et al.*, (2012) [16] and Chakra *et al.,* (2012) [10] also conclude that serum albumin and WCC levels are important mortality risk factors in patients with CDI, whilst presence of fever, haemoglobin/haematocrit level, diarrhoea severity, presence of renal disease, diabetes, cancer, or nasogastric tube use did not appear to be associated with mortality. This is consistent with findings in this study which also looked at these variables in relation to mortality and they were found not to be significant (data not shown). A recent publication has implicated that serum albumin, WCC and CRP are important prognostic variables for short term mortality in patients with CDI [17]. Other studies have found that a fall in serum albumin level was consistent with the onset of CDI [18] as well as being prognostic of mortality from CDI [11,17,19] and increased WCCs have also been implicated in other studies [11,19,20] as being prognostic of mortality in patients with CDI.

Whilst there is no certainty that an increase/decrease in clinical variables such as respiratory rate, WCC, CRP and serum albumin are alone due to CDI, as these patients are usually older, and have multiple co-morbidities, it is generally seen that these markers have usually returned to baseline levels before a later rise which occurs around the time of *C. difficile* diagnosis, which could be up to 1–2 weeks after cessation of antibiotic treatment for a previous condition. Thus, an acute rise/decline in these markers, around the time of infection diagnosis may be generally attributed to *C. difficile* infection and a combination of all these variables would prove useful as predictors of mortality in patients with CDI and warrants their inclusion in a prediction rule, as supported by others [11,17-20].

Other studies have suggested elevated urea as a marker of risk of mortality, however urea levels were not evaluated in this study as emphasis was placed on the % rise of creatinine from a baseline reading as specified by the Department of Health report [15]. Creatinine rise was not found to be a significant predictor of mortality in this study which might be attributed to particular emphasis being placed on maintaining hydration in the patients on the cohort ward, while in other clinical settings patients at CDI diagnosis are not often initially managed by a specialist. The role of urea will be re-evaluated in a prospective study to ensure that it does not add statistical strength to the prediction rule.

The (simplified) prediction rule derived in this study was significant at classifying those patients with increased mortality in the derivation cohort (AUC = 0.704; P < 0.001; 95% CI: 0.619-0.790) and has been made applicable to patient cohorts obtained from non-specialist environments, by its application to a validation cohort from the Bhangu *et al.* study [11]. It was shown to be consistent in classification of patients with increased mortality risk in the validation cohort (AUC = 0.653; P = 0.001; 95%; CI: 0.565-0.741), with that of the prediction rule developed in the actual study by Bhangu *et al.*, [11] (Table 4). This clearly shows the prediction rule was robust; even though a key variable was missing, when tested on a new data set, as the prediction rule remained statistically significant even though the AUC values were reduced. It is pertinent to note that scores of zero in both this study and that of Bhangu *et al.*[11] seem to still be associated with a higher mortality than that reflected in the CURB-65 study by Lim *et al.* (2003) [7] whereby scores of zero represent 0–0.5% mortality. The actual mortality in the Lim *et al.* (2003) [7] cohort was around 9.5%. The mortality rate for *C. difficile* cohorts used for this study were 24% (derivation cohort) and 38% (validation cohort). Therefore, clearly the base level of mortality for the prediction rule derived in this study will have a part to play in how discriminatory the test can be at lower levels of mortality. In the Lim *et al.,* (2003) [7] study the following mortality percentages are quoted for the CURB-65 score (in the validation cohort) 0 = 0%, 1 = 0%, 2 = 8.3%, 3 = 21.4%, 4 = 26.3, 5 = 33.3%. Analysis of this result showed that a score of 2 or less results in a lower risk of mortality than the mean (9.34%) and those above 2, an increased risk. In comparison, the prediction rule score for this study (in the validation cohort) is as follows; 0 = 20.9%, 1 = 37.1%, 2 = 54.3%, 3 = 66.7%. The same analysis holds true for this approach albeit for higher overall mortality rates, with points 0 and 1 resulted in lower than average (38%) risk of mortality and points 2 and 3 demonstrating increased risk. Thus, whilst the CURB-65 score is undoubtedly more discriminatory at the lower end of mortality than this proposed approach; the characteristics of the study data, which has a much higher mean risk of mortality, mean that the proposed rule is better at the higher end. This may perhaps be more helpful to someone who is first attending a patient presenting with CDI. Finally, it should be noted that although the zero score has an attendant mortality rate that is significantly higher than 0%, it is also significantly lower than the actual mean mortality in both derivation and validation cohorts (9.5% vs. 24% in the derivation cohort and 20.8% vs. 38% in the validation cohort). Nonetheless, this prediction rule would benefit from further prospective validation in the future.

## Conclusion

The prediction rule (Table 1) is based on sound statistical analysis of this large retrospective cohort study and validation in an independent cohort.

The use of a simple prediction rule in a clinical setting could facilitate the way in which clinicians are able to effectively manage a patient with CDI, perhaps prompting a different treatment regime if a high risk of mortality is identified. The prediction rule is simple; and uses only four variables as opposed to other studies, which use more variables [11], and could be used by non-specialists to consider mortality risk when assessing a patient presenting with CDI within or around 48 hours of diagnosis. It would also be useful in communication within teams and between teams, for example in discussion with microbiology doctors, as well as giving the patient and relatives information that is evidence-based. All findings in this study strengthen the evidence for establishment of this rule in a clinical setting.

## Competing interests

The authors declare that they have no competing interests.

## Authors’ contributions

SLM, RWT, EK, and RS obtained funding, conceived and designed the study. JB helped design the study. EB, JAHF, JB, AB, RS, SD, JH, CC, CM, HS, AG and RB acquired the data. EB and EK analysed and interpreted the data. EB and JAHF drafted the manuscript. EB, JAHF, EK, SLM, AB and RS critically revised the manuscript. EK and SLM supervised this study. EK is guarantor for this study. All authors read and approved the final manuscript.

## Funding

This work was funded by University of Exeter, Systems Biology Initiative, a small grants fund from the RD&E NHS Trust and The National Institute for Health Research (NIHR) Collaboration for Leadership in Applied Health Research and Care (CLAHRC) for the South West Peninsula (PenCLAHRC). This article presents independent research funded by the National Institute for Health Research (NIHR) Collaboration for Leadership in Applied Health Research and Care (CLAHRC) for the South West Peninsula. The views expressed in this publication are those of the author(s) and not necessarily those of the NHS, the NIHR or the Department of Health in England.

## Pre-publication history

The pre-publication history for this paper can be accessed here:

http://www.biomedcentral.com/1471-2334/13/316/prepub

## Supplementary Material

Additional file 1 Supplementary Material. Click here for file
