# Supplementary material for: Derivation and validation of a simple, accurate and robust prediction rule for risk of mortality in patients with Clostridium difficile infection
Source: BMC Infect Dis. 2013 Jul 12;13:316. doi: 10.1186/1471-2334-13-316 (PMC3733667; doi:10.1186/1471-2334-13-316)
Supplement: Additional file 1 — Supplementary Material. [file 1471-2334-13-316-S1.docx]

**Supplementary Material**

**Table 1**- Variables that were collated and analysed during the study.

| **Patient Demographics** | Age On Admission, Sex, |
| --- | --- |
| **Outcome History** | Length of stay, All-cause Mortality, **Severity of Infection** – Mild, Moderate, and Life threatening, Transfer To ICU After Positive result, colectomy for IBD, colectomy for *C. difficile* |
| **Physiological Measurements** | Systolic Blood Pressure, Diastolic Blood Pressure, Temperature, Respiratory Rate, White Blood Cell Count, Serum Albumin, C-Reactive Protein, Calprotectin, Creatinine % Raise from baseline, Neutrophils, Lactate, Platelets, Early Warning Score**,** Abdominal Pain On Examination**,** Abdominal Distension |
| **Co-morbidities** | **Type of co-morbidity-** Active Cancer, Acute Renal Failure, Acute Stroke, Chronic Obstructive Pulmonary Disease, Chronic Renal Failure Dialysis, Chronic Renal Failure Non-Dialysis, Dementia, Diabetes, Hypertension, Hypothyroidism, Inflammatory Bowel Disease, Ischaemic Heart Disease Or Congestive Cardiac Failure, Peripheral Vascular Disease, Post Gastrointestinal Surgery<Three Months, Stroke, Cancer type- solid, or haematological, Number of co-morbidities, co-morbidities present? |
| **Other variables** | **Admission reason**- Acute Medical, Community *C. difficile*, Elective Surgery, Emergency Surgery, Planned Procedure  Renal, Trauma. **Admission Residence**- Home, Long Term Care facility, Another Hospital, Acquisition Type- Hospital Acquired  Community Acquired, Community Acquired but IP in past 30 days. Nasogastric use, On PPI, **Type of PPI**-, Esomeprazole, Lansoprazole, Omeprazole, Rabeprazole. **Dose of PPI**-10 mg/day, 15 mg/day, 20 mg/day, 30 mg/day, 40 mg/day, 70 mg/day. Steroid use. H2 agonist use  Cancer chemotherapy use, Frequency of/and Bristol stool type (I-VII)  **Abdominal X-Ray Outcome-** Not Performed**,** Normal**,** Colitis**,** Toxic Mega colon**. Computed Tomography Scan outcome,** Not Performed  Colitis, Normal. **Flexible Sigmoidoscopy Outcome** -Not Performed, Normal, Colitis. Ribotype causing CDI |
| **Antibiotic history** | **Pre-*C. difficile* antibiotics** (by class)- Antiviral ,Beta Lactam Penicillin , Antifungal , Penicillin , Cephalosporin, Bacteriostatic, Macrolide,, Lincosamide, Sulfonomide, Lipopeptide, Tetracycline, Carbapenem, Amino Glycoside, Beta Lactam Carbapenems, Oxazolidinone, Nitroimidazole, Quinolone, Rifamycin  ***C. difficile* Treatments-** Glycopeptide**,** Fluconazole**,** Fusidic Acid**,** Intravenous Immunoglobulin, Intravenous Metronidazole  Intravenous Vancomycin, Oral Metronidazole, Oral Vancomycin, Per Rectum Vancomycin, Peritoneal Vancomycin, PR Metronidazole, Rifampicin. How may CDI treatments? How many pre-CDI antibiotics? |

**S1. Model Criteria**

A classification and regression tree criteria was chosen as a growing method. It uses a recursive partitioning method and builds classification and regression trees for predicting categorical predictor variables (classification). The model used an automatic maximum tree depth from the root node of 5, a minimum number of cases in a parent node of 10 which if split into further groups (child nodes), would contain a minimum number of 5 cases, to ensure outcome measurements were based on a sufficient amount of data. A misclassification cost of 3 was given for the prediction of death where the outcome was survival and a cost of 6 was given for the prediction of survival where the outcome was death, to account for the sample size bias.

**S2. Baseline Patient Demographics and Clinical Measurements**

**Site of Infection Acquisition**

67% (164) of cases admitted acquired *C. difficile* in hospital, 16% (40) of cases acquired *C. difficile* solely in the community, and 17% (41) cases acquired *C. difficile* in the community, but had been an in-patient in the last 30 days. 57.7% (123) of patients admitted were on gastric acid suppressing drugs of some sort and four patients were on cancer chemotherapy agents. Clinical measurements for each outcome group can be seen in Table 1.

**Patient Co-morbid Status**

A total of 15 co-morbidities were found in this cohort with patients having between 1-5 co-morbidities (median = 1) and 43 patients having no co-morbidities. Ischaemic heart disease or congestive cardiac failure (23.7%) was the most prevalent co-morbidity, followed by active cancer (14%) and diabetes (12.4%), there were only four cases of inflammatory bowel disease within this cohort.

**Pre and Post CDI Treatment Therapies**

A total of 34 antibiotics types were prescribed to this patient cohort, with most patients being prescribed from 1- 8 (median = 2) antibiotics during the length of stay and four patients having no antibiotics prior to onset of CDI. The 34 antibiotics could be grouped into 20 classes and the main class of antibiotic prescribed were the penicillin’s. No antibiotic was a clearly prescribed more than any other. This response is not able to distinguish which patient was prescribed multiple antibiotics

A total of 11 CDI treatment options were indentified, with patients receiving 1-5 treatments (median =1) and 10 patients receiving no treatment, either due to palliative care or other reasons. Oral metronidazole (45.1%) and oral vancomycin (35.6%) accounted for most of the treatment chosen but other methods included intravenous metronidazole and vancomycin, fluconazole, fusidic acid and rifampicin.

**Table 2** – Baseline characteristics of cohort according to the outcome group of all-cause mortality

|  | **Patient survived** | | | | **CDI related mortality** | | | | **Non-CDI related mortality** | | | |
| --- | --- | --- | --- | --- | --- | --- | --- | --- | --- | --- | --- | --- |
|  | Mean | N | s.d | Median | Mean | N | s.d | Median | Mean | N | s.d | Median |
| **Age on Admission (yrs)** | 76.8 | 194.0 | 14.1 | 80.0 | 82.1 | 27.0 | 9.6 | 83.0 | 80.9 | 24.0 | 9.3 | 82.0 |
| **Length of Stay in hospital (days)** | 39.0 | 194.0 | 28.8 | 31.0 | 37.6 | 27.0 | 35.5 | 29.0 | 36.6 | 24.0 | 27.1 | 24.5 |
| **Pulse (beats per min)** | 88.1 | 184.0 | 15.0 | 86.0 | 85.0 | 26.0 | 17.4 | 87.0 | 95.5 | 24.0 | 22.6 | 94.0 |
| **Systolic Blood pressure (mm/Hg)** | 124.5 | 185.0 | 22.3 | 124.0 | 115.2 | 26.0 | 28.6 | 113.0 | 121.0 | 24.0 | 19.9 | 118.0 |
| **Diastolic blood pressure (mm/Hg)** | 69.2 | 184.0 | 13.2 | 70.0 | 63.1 | 26.0 | 13.5 | 60.0 | 66.5 | 24.0 | 13.5 | 60.0 |
| **Temperature (°C)** | 36.8 | 183.0 | 0.6 | 36.8 | 36.6 | 26.0 | 0.6 | 36.6 | 37.0 | 23.0 | 0.8 | 37.0 |
| **Respiratory rate (breaths per minute)** | 17.1 | 178.0 | 3.7 | 16.0 | 19.3 | 26.0 | 3.7 | 18.5 | 19.7 | 23.0 | 5.4 | 18.0 |
| **White Blood Cell Count (x10^3^mcL)** | 13.0 | 193.0 | 7.5 | 11.0 | 22.7 | 26.0 | 22.1 | 17.0 | 21.3 | 24.0 | 33.9 | 10.5 |
| **Percent Rise In Creatinine from Baseline** | 31.6 | 193.0 | 51.8 | 19.0 | 51.3 | 26.0 | 70.4 | 20.6 | 21.1 | 23.0 | 31.1 | 11.7 |
| **CRP Levels (mg/L)** | 95.2 | 181.0 | 74.2 | 79.0 | 174.0 | 23.0 | 86.0 | 180.0 | 134.3 | 24.0 | 91.9 | 116.0 |
| **Neutrophil Count (cells/ul)** | 10.7 | 193.0 | 8.6 | 8.0 | 14.4 | 26.0 | 8.4 | 14.0 | 12.7 | 22.0 | 10.2 | 8.5 |
| **Platelet Levels (mcL)** | 316.8 | 193.0 | 152.6 | 301.0 | 266.7 | 26.0 | 129.4 | 290.5 | 269.1 | 24.0 | 112.3 | 273.5 |
| **Serum Albumin Levels (g/L)** | 31.1 | 165.0 | 5.4 | 31.0 | 25.6 | 20.0 | 5.0 | 24.5 | 27.4 | 19.0 | 5.5 | 27.0 |

N- Number of cases in sample

**S3. Statistical Analysis- Pair wise Comparison Analysis**

**Parametric tests**

One-way ANOVA tests for differences between means of groups with respect to the outcome measure all cause mortality (survived; CDI related mortality and non-CDI related mortality) were used for normally distributed interval data. The mean serum albumin levels for those who survived as an outcome (31.28 g/L), those whose death was related to CDI (25.60g/L) and those whose death was not attributed to CDI (27.37 g/L) were significantly different at P=0.00 (F=12.45; df=178).

**Non- Parametric Tests**

Independent samples K-median tests were used for variables which were non-normally distributed with respect to the outcome measure.

Pair wise comparison of all groups revealed that there was a statistical difference in median respiratory rate between the survival group (16 resps/min) and the group whose death was related to CDI (18.5 resps/min) (p=0.005) but not the group whose death was not related to CDI (18).

Pair wise comparison of all groups revealed that there was a statistical difference in median CRP levels between the survival group (79 mg/L) and the group whose death was related to CDI (180 mg/L) (p=0.0025) but not the group whose death was not related to CDI (116 mg/L).

Pair wise comparison of all groups revealed that there was a statistical difference in median white cell count levels between the survival group (11 x10^3^ mcL) and the group whose death was related to CDI (17 x10^3^mcL) (p=0.002) but not the group whose death was not related to CDI (10.5 x10^3^mcL).

**S4. Multinomial Logistic Regression Parameter Analysis**

**Table 3 -** Parameter estimates for the all cause mortality outcome measures CDI related mortality and non-CDI related mortality verses the survival outcome

| **All-cause Mortality** | | **β** | **Std. Error** | **Wald** | ***df*** | **Sig.** | **Exp(β)** | **95% Confidence Interval for Exp(β)** | |
| --- | --- | --- | --- | --- | --- | --- | --- | --- | --- |
|  |  |  |  |  |  |  |  | **Lower Bound** | **Upper Bound** |
| **CDI related mortality** | **Intercept** | -1.217 | 2.119 | .330 | 1 | P=0.566 |  |  |  |
|  | **Respiratory Rate** | .200 | .068 | 8.791 | 1 | P=0.003 | 1.222 | 1.070 | 1.395 |
|  | **Albumin** | -.221 | .067 | 10.850 | 1 | P=0.001 | 0.801 | 0.703 | 0.914 |
|  | **White Blood Cell Count** | .045 | .020 | 5.016 | 1 | P=0.025 | 1.046 | 1.006 | 1.088 |
|  | **C-Reactive Protein** | .009 | .004 | 5.388 | 1 | P=0.020 | 1.009 | 1.001 | 1.017 |
| **Non-CDI related mortality** | **Intercept** | -.967 | 1.941 | .248 | 1 | P=0.618 |  |  |  |
|  | **Respiratory Rate** | .171 | .063 | 7.322 | 1 | P=0.007 | 1.186 | 1.048 | 1.342 |
|  | **Albumin** | -.170 | .059 | 8.174 | 1 | P=0.004 | 0.844 | 0.751 | 0.948 |
|  | **White Blood Cell Count** | .005 | .030 | .024 | 1 | P=0.877 | 1.005 | 0.947 | 1.066 |
|  | **C-Reactive Protein** | .007 | .004 | 3.196 | 1 | P=0.074 | 1.007 | 0.999 | 1.015 |

-2Log Likelihood =166.3 (χ^2^= 58.6, *df*=8, P=0.000), Nagelkerke R^2^=40.5, Pearson Chi-square Test; P=0.996.

**S5. Decision Tree classification**

**Table 4**- Classification of survival and death groups for the test data for the outcome all-cause mortality.

| **Observed (N)** | **Predicted** | | | |
| --- | --- | --- | --- | --- |
|  | **Survived** | **CDI related mortality** | **Non-CDI related mortality** | **Percent Correct** |
| **Survived** | 121 | 3 | 0 | 97.6% |
| **CDI related mortality** | 9 | 9 | 1 | 47.4% |
| **Non-CDI related mortality** | 12 | 6 | 0 | .0% |
| **Overall Percentage** | 88.2% | 11.2% | .6% | 80.7% |
